# Flow-Induced Shear Stress Combined with Microtopography Inhibits the Differentiation of Neuro-2a Cells

**DOI:** 10.3390/mi16030341

**Published:** 2025-03-16

**Authors:** Eleftheria Babaliari, Paraskevi Kavatzikidou, Dionysios Xydias, Sotiris Psilodimitrakopoulos, Anthi Ranella, Emmanuel Stratakis

**Affiliations:** 1 Foundation for Research and Technology—Hellas (F.O.R.T.H.), Institute of Electronic Structure and Laser (I.E.S.L.), Vasilika Vouton, 70013 Heraklion, Greece; ebabaliari@iesl.forth.gr (E.B.); ekavatzi@iesl.forth.gr (P.K.); dion.xydias@iesl.forth.gr (D.X.); sopsilo@iesl.forth.gr (S.P.); 2 Department of Materials Science and Technology, University of Crete, 70013 Crete, Greece; 3 Department of Physics, University of Crete, 70013 Crete, Greece

**Keywords:** neuronal differentiation, microfluidics, microfluidic flow, shear stress, laser fabrication, topography

## Abstract

Considering that neurological injuries cannot typically self-recover, there is a need to develop new methods to study neuronal outgrowth in a controllable manner in vitro. In this study, a precise flow-controlled microfluidic system featuring custom-designed chambers that integrate laser-microstructured polyethylene terephthalate (PET) substrates comprising microgrooves (MGs) was developed to investigate the combined effect of shear stress and topography on Neuro-2a (N2a) cells’ behavior. The MGs were positioned parallel to the flow direction and the response of N2a cells was evaluated in terms of growth and differentiation. Our results demonstrate that flow-induced shear stress could inhibit the differentiation of N2a cells. This microfluidic system could potentially be used as a new model system to study the impact of shear stress on cell differentiation.

## 1. Introduction

Nerve injuries in adults represent a significant clinical challenge, greatly impacting patients’ quality of life [1]. These injuries contribute to around 6.8 million deaths annually and impact more than 1 billion people globally [2]. They are associated with various disorders including traumatic brain and spinal cord injuries, stroke, and neurodegenerative diseases [3]. Neurological disorders, which affect both the central nervous system (CNS) and the peripheral nervous system (PNS), such as Alzheimer’s and Parkinson’s disease, have been on the rise, posing a significant threat to the well-being of those affected, particularly older adults, as well as their families. While these disorders can result from factors like selective cell loss in the CNS, their precise origins and triggers remain unknown [4,5].

The nervous system is divided into two main components: the CNS and the PNS. While the CNS cannot typically regenerate after an injury, the PNS possesses inherent regenerative capabilities [6]. However, peripheral nerves can only regenerate on their own if the injury does not result in a significant gap in the nerve. In most cases, this type of regeneration is rarely functional due to the disorganized and mismatched nature of spontaneous axon regeneration, leading to insufficient recovery of nerve function and limitations in musculoskeletal capability [1].

Autologous nerve grafts (autografts) remain the gold standard for treating peripheral injuries but have drawbacks like additional surgeries, donor site complications, functional nerve loss, and extended surgical time [1,6]. This has led to tissue engineering becoming a key field in developing graft substitutes. The goal is to replicate the structural characteristics and cues of the extracellular matrix (ECM) to encourage natural cell behavior in artificial environments [7]. As a result, scaffold development has gained scientific interest, with methods like femtosecond laser structuring, soft lithography, photolithography, and electrospinning being effective in mimicking ECM structures in vitro [8,9,10]. Previous research has reported the main fabrication methods, the resulting micro/nanostructured substrates, and their corresponding advantages and disadvantages [2,11,12].

Among such methods, femtosecond laser structuring emerges as a simple, fast, single-step, and non-contact process, applicable to a wide range of materials (metals, ceramics, polymers). Additionally, it has the capability to texture materials on their surfaces and etch deeper regions within the material’s bulk, enabling the creation of structures with complex geometries [12,13]. Materials that have been laser-patterned with structures like pillars, grooves, micro- and nano-cones [14], as well as channels are extensively utilized as cell culture platforms, aiming to explore the effect of topographical cues on diverse cellular responses [15]. Micro- and/or nano-structured substrates significantly influence neural cell growth and differentiation by providing physical cues that guide cellular behavior. At the nanoscale, features such as growth cone filopodia, present at the tips of extending axons, are highly responsive to topographic signals, directing axon development along natural pathways. These cues, including aligned ECM patterns and surface textures, impact crucial processes in neural cells, such as adhesion, polarization, migration, and alignment. Neurons, particularly during development, rely on these topographical features to navigate and extend their axons, ensuring proper network formation. Additionally, the topography of the material influences the reorganization of the cytoskeleton in neural cells, as well as controlling their directionality and growth [16].

Apart from structural characteristics, the mechanical environment (e.g., mechanical stresses) provided by the ECM should not be neglected [13]. Flow-induced shear stress can be exerted on cells in vitro through microfluidic devices. In contrast to conventional static cultures, microfluidic cell cultures enable precise microenvironment control. This includes adjustments in parameters such as the flow rate, pH, and oxygen (O_2_) levels, all of which play a crucial role in influencing the biochemical and mechanical factors within a cell, thereby impacting its functionality [17]. Particularly, microfluidic flow cell cultures replicate the in vivo cellular environment by simulating factors such as physiological fluid flow, constant nutrient supply, mechanical stimulation from fluid shear forces, and efficient waste removal [16,18]. Flow-induced shear stress influences mechanoreceptors, including ion channels and focal adhesions, triggering responses such as nitric oxide production, intracellular calcium signaling, and cytoskeletal remodeling. When applied at discrete points, shear stress transmits through cytoskeletal structures, altering cell shape and modulating intracellular pathways. As a key component of the cellular environment, shear stress significantly impacts cellular signal transduction and behavior. It is known that flow-induced shear stress affects cytoskeletal reorganization and cellular function across various cell types. In neurons, both the soma and neurites respond to mechanical stimuli via the cytoskeleton, which plays a crucial role in axon regeneration [16].

Many studies have reported the effect of dynamic culture conditions on the behavior of neuronal and/or glial cells [19,20,21,22,23]. Specifically, Kim et al. [19] used a microfluidic chip-generated growth factor gradient system exposing neural stem cells (NSCs) to a concentration gradient of known growth factors (GFs). The results indicated that the proliferation and differentiation of NSCs were directly influenced by the concentration gradient of GFs. Futai et al. [20] fabricated a one-layer H-shaped microfluidic channel, composed of a thin microchannel with a high aspect ratio (~2 μm thickness and 0.5–1 aspect ratio), to generate concentration gradients. They employed a long-lasting nerve growth factor (NGF) concentration gradient to investigate the elongation of axons in primary dorsal root ganglion (DRG) neuronal cells. The findings demonstrated that axon elongation occurred in the direction of the NGF concentration gradient during the 96-h cultivation period. Furthermore, Bhattacharjee and Folch [21] developed a microfluidic device comprising 1024 biochemical gradient generators. Every single generator captured a single neuron, facilitating the examination of growth dynamics and axon guidance in primary hippocampal neurons when exposed to biochemical signals. The microfluidic chip generated a consistent and stable gradient while exerting minimal shear stress on the cultured surface (100 μL/h), with a shear stress value lower than 1.2 × 10^−3^ Pa. Utilizing this platform, it was shown that the guidance of hippocampal axons depended on both the concentration and angle of incidence of the netrin-1 gradient. Particularly, concerning concentration, they observed that hippocampal growth cones located closer to the netrin-1 source (where the concentration was high) showed a strong attraction, while those farther away (at lower concentrations) were repelled. As for the angle of incidence, they found that growth cones oriented away from the gradient axis (between 90° and 135°) redirected toward the netrin-1 source, while those aligned with the gradient (less than 45°) were strongly repelled. Park et al. [22] fabricated a two-dimensional microfluidic system to examine the effect of shear stress under continuous flow conditions (10^−4^ Pa, 10^−3^ Pa) on radial glial cells (RGCs). The results revealed that flow shear stress may trigger the activation of mechanosensitive Ca^+2^ channels, resulting in a significant increase in the proliferative ability of RGCs when subjected to increased shear stress values. Finally, Ristola et al. [23] fabricated an innovative microfluidic cell culture system utilizing polydimethylsiloxane (PDMS), incorporating open compartments, specifically engineered for studying neuron-oligodendrocyte myelin interactions in vitro. The results demonstrated successful co-culturing of primary rat DRG neurons and oligodendrocytes within the microfluidic system, facilitating time-lapse imaging. Findings further revealed successful neurite-oligodendrocyte interactions and contacts, along with the deposition of myelin segments organized in a highly ordered arrangement within the microfluidic system.

However, few studies investigated the combined effect of microfluidic flow and topography on neuronal outgrowth [24,25,26,27]. In particular, Hesari et al. [24] developed a hybrid microfluidic system comprising a PDMS microchip and poly(lactic-co-glycolic acid) (PLGA) nanofiber-based substrate to induce the differentiation of human-induced pluripotent stem cells (hiPSCs) into neurons. Their findings demonstrated an upregulation in the gene expression of β-tubulin III, a specific neuronal marker, alongside a downregulation in the gene expression of the glial fibrillary acidic protein (GFAP), a classic astrocyte marker. This suggested the potential of this hybrid microfluidic system for enhancing neuronal differentiation. Kim et al. [25] designed a fluid flow system to investigate how mechanical stimulation affects pheochromocytoma (PC12) cells seeded on microfiber-based substrates. Their results revealed that shear stress affected both the orientation and length of neurons along the microfibers. Moreover, Jeon et al. [26] investigated how the combined effect of topography and flow-induced shear stress influences the neuronal differentiation of human mesenchymal stem cells (hMSCs). They subjected cells to various shear stresses on a PDMS substrate featuring micrometric grooves. The results showed that shear stresses influenced the β-tubulin III expression, synaptophysin, and microtubule-associated protein 2 (MAP2), along with intracellular calcium levels. Additionally, alignment was confirmed. An increase in neurite length was observed on the seventh day, but a significant decrease occurred by the tenth day. It is important to note that in both studies [25,26], the induced flow was not continuous but applied for only a few hours per culture day. Our group, in a previous study, investigated the combined effect of shear stress and topography on Schwann (SW10) cells’ behavior under dynamic culture conditions attained through continuous flow [27]. For this purpose, we developed a precise flow-controlled microfluidic system with custom-designed chambers containing laser-structured PET microgrooved substrates [28]. The MGs were placed parallel or perpendicular to the flow direction, and the response of SW10 cells was evaluated based on their growth, elongation, and orientation. The findings revealed that depending on the relation of the flow direction and topographical features, wall shear stress gradients acted either synergistically or antagonistically with topography, influencing the guided morphological response of cells. The aforementioned studies emphasize the need to develop biomimetic in vitro cell culture systems that more accurately replicate the in vivo microenvironment for investigating neuronal outgrowth.

Τhis work aims to present a first study of the combined effect of shear stress and topography on the growth and differentiation of N2a cells under continuous flow conditions. N2a is a mouse neural crest-derived cell line that has been extensively used as a model system to study proliferation, neuronal differentiation, neurite outgrowth, signaling pathways, and cytotoxicity [29,30,31,32,33,34]. For this reason, we used the precise flow-controlled microfluidic system featuring custom-designed chambers that integrate laser-microstructured PET culture substrates containing MGs [27,28]. PET is one of the most common polymers widely used for cell culturing and biomedical applications [35,36]. Specifically, chemically inert PET biomaterials, combined with different coatings, are used in sutures, vascular grafts, surgical meshes, scaffolds, heart valves, urinary, and bloodstream catheters due to their biocompatibility and excellent mechanical strength as well as resistance [35,36,37]. The MGs were positioned parallel to the direction of the flow and the response of N2a cells was evaluated in terms of growth and differentiation. Our findings revealed that flow-induced shear stress may inhibit the differentiation of N2a cells. Hence, the biomimetic microfluidic system presented here could be potentially used as a new model system to study the role of shear stress on cell differentiation.

## 2. Materials and Methods

### 2.1. Development of the Microfluidic System

**Figure 1:**
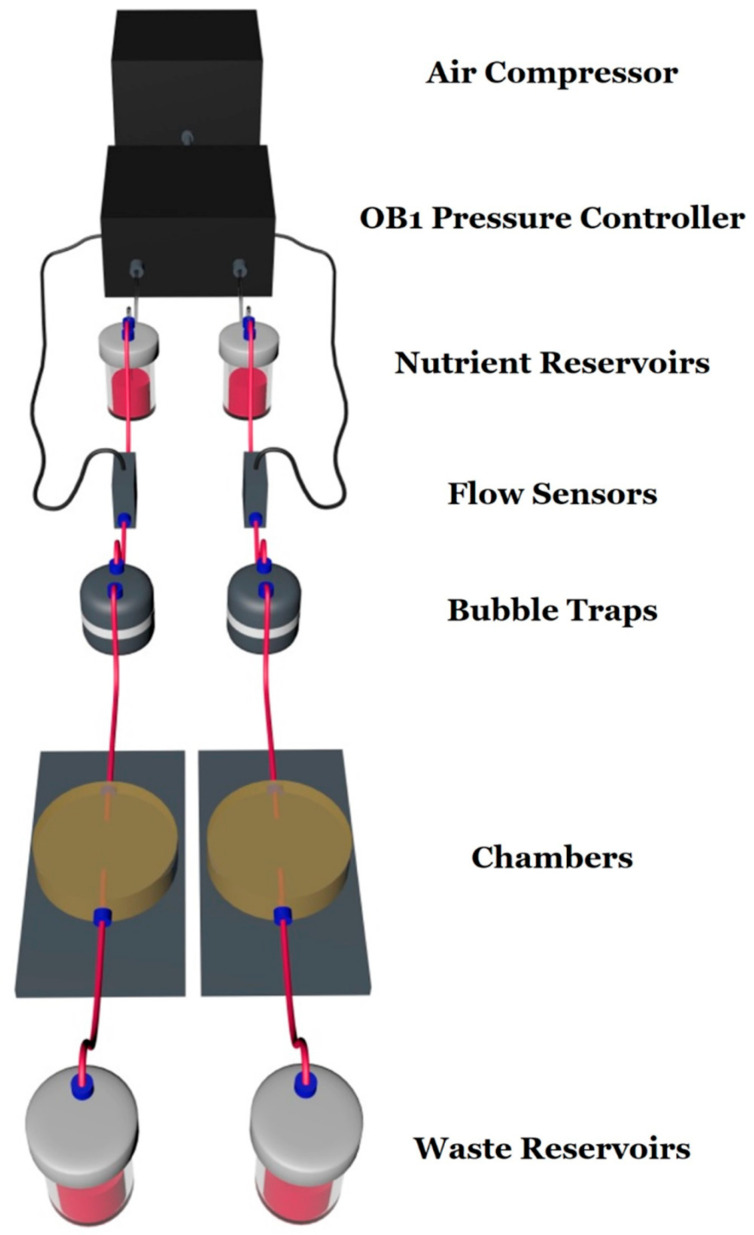
Schematic illustration of the custom-designed microfluidic system. The system consists of an air compressor and an OB1 pressure controller connected with nutrient reservoirs, flow sensors, bubble traps, chambers containing the cells and the laser-microstructured substrates, and waste reservoirs.

The microfluidic system [27] consists of an air compressor (Durr Technik, Melville, NY, USA) and an OB1 pressure controller (Elveflow, Paris, France) connected through silicon tubing to the nutrient reservoirs (Elveflow, Paris, France) (Figure 1). Afterward, the nutrient (in this case culture medium), through poly(tetrafluoroethylene) (PTFE) tubing with an interior diameter of 0.5 mm, moves to the flow sensors (Elveflow, Paris, France), to the bubble traps (Elveflow, Paris, France), to the custom-made geometry chambers (Ebers, Zaragoza, Spain) containing the laser-microstructured substrates with the cells, and finally to the waste reservoirs (Elveflow, Paris, France). The laser-microstructured substrates were placed parallel to the direction of the flow inside the chambers. The chambers are composed of polysulfone upper lids for cell loading (6 mm length, 6 mm width, 13 mm height) and for fluid flow across the cells and the laser-microstructured substrates (11 mm length, 6 mm width, 150 μm height). The experiments were performed under continuous flow conditions. The chambers and the waste reservoirs were placed inside a 5% carbon dioxide (CO_2_) incubator at 37 °C for the whole duration of the dynamic culture experiments.

### 2.2. Fabrication of Laser-Microstructured Polyethylene Terephthalate (PET) Substrates

The microstructured substrates were developed by employing ultrafast laser direct writing on PET coverslips designated for cell cultures (Sarstedt, Numbrecht, Germany). This method, characterized by its simplicity, low cost, and effectiveness, allows precise control over the substrate’s topography [2]. Specifically, a Yb:KGW laser source with a wavelength of 1026 nm, a repetition rate of 1 kHz, and a pulse duration of 170 fs was utilized for this purpose. The laser-microstructured substrates were fabricated using a constant fluence of 11.9 J/cm^2^ and a scan velocity of 7 mm/s. The resulting patterned area was 3 mm × 3 mm with MG width and spacing equal to 28.68 ± 0.47 μm and 28.76 ± 0.50 μm, respectively. The geometry and dimensions of the MGs were selected based on our previous studies [27,28], which showed that they provided a favorable environment for neural growth.

### 2.3. Morphological and Geometrical Characterization of Laser-Microstructured Polyethylene Terephthalate (PET) Substrates by Scanning Electron Microscopy (SEM)

The laser-microstructured substrates were sputter-coated with a 15 nm gold layer (Baltec SCD 050, BAL-TEC AG, Balzers, Liechtenstein) and observed using a scanning electron microscope (JEOL JSM-6390 LV, Jeol USA Inc., Peabody, MA, USA) with an acceleration voltage of 15 kV. Fiji ImageJ (v1.52i), an image processing software, was utilized for the analysis of the geometrical characteristics of the MGs as described in reference [28]. The aspect ratio of the MGs (A) was determined by calculating the ratio of their depth (d) to width (w) (A = d/w). The roughness ratio (r) was calculated as the ratio of the actual, unfolded surface area of the MGs to the total irradiated area [r = 1 + (2 d/w)].

### 2.4. Static and Dynamic Cell Cultures

Neuro-2a (N2a) cells (ATCC-LGC, Rockville, MD, USA) are a fast-growing mouse neuroblastoma cell line with neuronal and amoeboid stem cell morphology isolated from brain tissue. N2a cells were grown in cell culture flasks using culture medium [Dulbecco’s Modified Eagle’s Medium (DMEM) (Invitrogen, Grand Island, NY, USA) supplemented with 10% Fetal Bovine Serum (FBS) (Biosera, Sussex, UK) and 1% antibiotic solution Pen-Strep (PS) (Gibco, Invitrogen, Kalsruhe, Germany)] in a 5% CO_2_ incubator (Thermo Scientific, OH, USA) at 37 °C.

Before conducting any experiment, the laser-microstructured substrates, the chambers, the reservoirs, and the bubble traps underwent ultraviolet (UV) sterilization. Then, the substrates were placed in sterile wells of 24-well plates (Sarstedt, Numbrecht, Germany) for static cultures and inside the chambers of the microfluidic system for dynamic cultures. Planar PET coverslips for cell cultures (Sarstedt, Numbrecht, Germany), referred to as PET-Flat, were utilized as the control samples.

Prior to any culture, the substrates were coated with a 15 μg/mL laminin solution (Sigma-Aldrich, St. Louis, MO, USA) to enhance cell adhesion. For the static cultures, 3 × 10^4^ cells/1.9 cm^2^ (i.e., ~1.58 × 10^4^ cells/cm^2^) were seeded on the planar PET coverslips and the laser-microstructured substrates and were cultured for 3 days. For the dynamic cultures, 3 × 10^4^ cells/0.36 cm^2^ (i.e., 8.33 × 10^4^ cells/cm^2^) were seeded on the planar PET coverslips and the laser-microstructured substrates, placed inside the chambers, and kept at rest overnight in a 5% CO_2_ incubator at 37 °C. Static cultures were seeded at 3 × 10^4^ cells/1.9 cm^2^ (i.e., ~1.58 × 10^4^ cells/cm^2^) to allow for proper growth with diffusion-based nutrient exchange. In dynamic cultures, 3 × 10^4^ cells/0.36 cm^2^ (i.e., 8.33 × 10^4^ cells/cm^2^) were used to accommodate both the improved nutrient delivery from media flow and potential cell detachment, ensuring optimal growth conditions. The following day, the continuous perfusion was initiated and continued for a duration of 2 days. Based on the results of our previous work [27], flow rates of 15 and 30 μL/min were used. At higher flow rates, cells detached from the substrates.

The equation for estimating the mean velocity, $\bar{u}$, in the microfluidic system is given by $\bar{u}$ = (4Q)/(π$d^{2}$) [38], where Q represents the flow rate and d the tubing diameter (d = 0.5 mm).

The equation σ = (6ηQ)/(b$h^{2}$) is used to calculate the shear stress exerted on the cell layer [39,40], where η represents the viscosity of the nutrient (η~0.01 g${cm}^{-1}s^{-1}$ [41,42]), Q the flow rate, b the width of the upper lid of the chamber for fluid flow (b = 6 mm), and h the height of the upper lid of the chamber for fluid flow (h = 150 μm).

Table 1 presents the flow rates along with the associated mean velocity and shear stress in the microfluidic system.

Considering that the differentiation of N2a cells is equally induced by serum deprivation and/or the addition of cyclic AMP (cAMP) [43], we studied both differentiation conditions. For the cell differentiation experiments, under static conditions, the culture medium was substituted with serum-free [without (w/o) FBS] DMEM (Invitrogen, Grand Island, NY, USA) containing 1% antibiotic solution PS (Gibco, Invitrogen, Kalsruhe, Germany) or serum-free (w/o FBS) DMEM (Invitrogen, Grand Island, NY, USA) containing 1% antibiotic solution PS (Gibco, Invitrogen, Kalsruhe, Germany) and 300 μM cyclic AMP (cAMP) (Sigma-Aldrich, St. Louis, MO, USA) after 24 h of incubation. Under dynamic conditions, the culture medium was substituted with serum-free (w/o FBS) DMEM (Invitrogen, Grand Island, NY, USA) containing 1% antibiotic solution PS (Gibco, Invitrogen, Kalsruhe, Germany). Thus, for differentiation, cells were cultured in serum-free (w/o FBS) DMEM containing 1% antibiotic solution PS to support differentiation by removing growth factors that favor proliferation or/and in serum-free (w/o FBS) DMEM containing 1% antibiotic solution PS and 300 μM cAMP to promote neural differentiation, as cAMP enhances differentiation processes. DMEM with 10% FBS and 1% antibiotic solution PS was used as the standard culture medium to support cell survival and proliferation rather than differentiation.

For each case investigated, a series of three independent experiments were performed.

### 2.5. Morphological Characterization of N2a Cells by Scanning Electron Microscopy (SEM)

The adhesion of the cultured cells on the planar PET coverslips and the laser-microstructured substrates was examined by scanning electron microscopy (SEM). Specifically, the planar PET coverslips and the laser-microstructured substrates seeded with the cells were removed from the incubator on the third day of culture, underwent two washes using 0.1 M sodium cacodylate buffer (SCB), and were subsequently fixed with a mixture of 2% glutaraldehyde (GDA) and 2% paraformaldehyde (PFA) (Sigma-Aldrich, St. Louis, MO, USA) in 0.1 M SCB for a duration of 30 min. Following this fixation, the samples underwent two additional washes with 0.1 M SCB and were subjected to dehydration using progressively increasing concentrations of ethanol (ranging from 30% to 100%). Subsequently, they were dried using a critical point drier (Baltec CPD 030, BAL-TEC AG, Balzers, Liechtenstein), coated with a 15 nm layer of gold using a sputter coater (Baltec SCD 050, BAL-TEC AG, Balzers, Liechtenstein) and finally examined under a scanning electron microscope (JEOL JSM-6390 LV, Jeol USA Inc., Peabody, MA, USA) at an accelerating voltage of 15 kV.

For each case investigated, a series of three independent experiments were performed.

### 2.6. Immunocytochemical Assays

A series of cell cultures on planar PET coverslips and laser-microstructured substrates were stained to study cell growth and differentiation.

To perform immunofluorescence staining, the samples were removed from the incubator and washed twice with phosphate buffered saline (PBS) after 3 days of culture. Afterward, they were fixed with 4% PFA for 15 min and permeabilized with 0.1% Triton X-100 (Sigma-Aldrich, St. Louis, MO, USA) in PBS for 5 min. The non-specific binding sites were blocked with 2% bovine serum albumin (BSA) (Sigma-Aldrich, St. Louis, MO, USA) in PBS for 30 min.

The double-stranded helical DNA of the cell nucleus and the F-actin of the cytoskeleton were stained with 4′,6-Diamidino-2-Phenylindole (DAPI) and phalloidin, respectively. Actin serves as the major cytoskeletal protein in the majority of cells. It is highly conserved and participates in diverse functional and structural roles [44]. In this case, actin is present in the form of actin filaments (F-actin) and is stained using a specific phalloidin (Alexa Fluor^®^ 680 Phalloidin). The samples underwent a 2-h incubation at room temperature using Alexa Fluor^®^ 680 Phalloidin (Invitrogen, Thermo Fisher Scientific, Waltham, MA, USA) (1:250 in PBS-BSA 1%) for F-actin staining. Then, they were washed with PBS and mounted on coverslips with DAPI (Molecular Probes by Life Technologies, Carlsbad, CA, USA) for nuclei staining.

The TUJ1 (Merck, Germany), a marker of neuronal differentiation, was applied to the samples (1:400 in PBS-BSA 1%) and they were incubated for 1 h at room temperature. Afterward, they were washed with PBS and incubated for 1 h at room temperature with anti-mouse CF^®^488A (Biotium, San Francisco, CA, USA) (1:400 in PBS-BSA 1%). Finally, they were washed with PBS and mounted on coverslips with DAPI (Molecular Probes by Life Technologies, Carlsbad, CA, USA) for nuclei staining.

Cell imaging was conducted utilizing a Leica SP8 inverted scanning confocal microscope (Leica Microsystems, Germany). The objective of ×40 was used. To obtain images of the cells both on the upper surface and inside the MGs, the z-stack of the confocal microscope was used.

For each case investigated, a series of three independent experiments were performed.

### 2.7. Live Cell Imaging Assay

The fluorescent dye acridine orange (AO) (Thermo Scientific, OH, USA) was used to perform the imaging of the live cells cultured on the planar PET coverslips and the laser-microstructured substrates in order to assess cell differentiation. AO stain is a cell-permeant vital dye that binds to nucleic acids. In particular, the samples were removed from the incubator and washed with PBS after 3 days of culture. Afterward, they incubated with 8 μg/mL AO solution in culture medium for 5 min at 37 °C. Then, they were washed with fresh pre-warmed culture medium to remove excess dye. Finally, they were transferred to glass-bottomed petri dishes (Thermo Scientific, OH, USA) to be observed under the custom-built inverted laser raster-scanning non-linear microscope. For each case investigated, a series of three different experiments were performed.

### 2.8. Non-Linear Microscope

**Figure 2:**
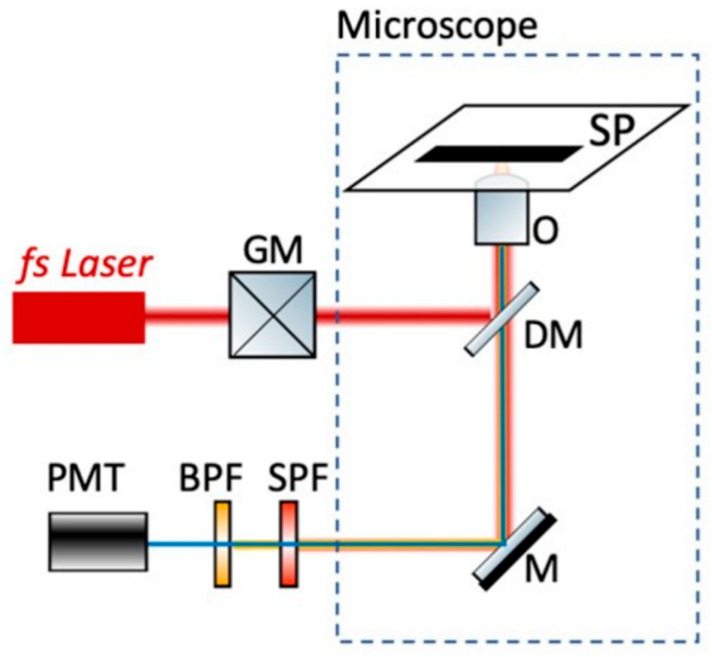
Schematic representation of the experimental setup used for two-photon fluorescence (2p-F) imaging microscopy of acridine orange (AO) in live cells. Abbreviations: GM, galvanometric mirrors; DM, dichroic mirror that reflects wavelengths longer than 700 nm and lets pass wavelengths shorter than 700 nm; O, objective lens; SP, sample plane; SPF, short-pass filter that lets pass wavelengths shorter than 680 nm; BPF, band-pass filter that lets pass wavelengths in the range 562 ± 20 nm; PMT, photomultiplier tube.

By using a femtosecond (fs) laser source, we excite two-photon fluorescence (2p-F) from the fluorescent dye AO in live cells. Our custom-built multiphoton microscope (Figure 2) is based on a 1030 nm fs laser (Flint, Light Conversion, Vilnius, Lithuania), which passes through a pair of galvanometric mirrors (6215 H, Cambridge Technology, Worthington, MN, USA) before entering into an inverted microscope (Axio Observer Z1, Carl Zeiss, Jena, Germany) [45]. The beam is then reflected by a short-pass dichroic mirror (FF700-SDi01, Semrock, Rochester, NY, USA) placed at the turret box of the microscope and is focused into the sample with a long working distance 50× 0.42NA objective-lens (Mitutoyo Plan Apochromat, Sakado, Japan). The emitted fluorescence signals are collected by the same objective and are first filtered by a short-pass filter (FF01-680/SP, Semrock) to ensure that no laser light is propagating to the detector. Then, a band-pass filter (FF01-562/40-25, Semrock) follows, which allows passing the wavelengths in the range of 562 ± 20 nm, before reaching a detector, based on a photomultiplier tube (PMT) (H9305-04, Hamamatsu, Japan). Coordinate motion of the galvanometric mirrors and the detector for image acquisition is performed using custom-built Labview 13.00 (National Instruments, Austin, TX, USA) software.

## 3. Results

### 3.1. Morphological Characterization of Laser-Microstructured Polyethylene Terephthalate (PET) Substrates

**Figure 3:**
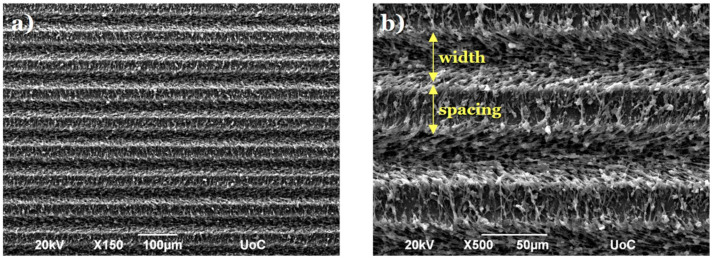
(**a**) Top-view scanning electron microscopy (SEM) images of the polyethylene terephthalate microgrooved (PET-MG) substrates. (**b**) Higher magnification image of Figure (**a**). The width and spacing of the MGs are indicated directly in the SEM image.

The laser-structured microgrooved substrates were morphologically characterized by SEM. Figure 3 presents top-view SEM images, revealing that the MG spacing and width (w) measured 28.76 ± 0.50 μm and 28.68 ± 0.47 μm, respectively. The depth (d) was found to be 8.87 ± 0.44 μm, resulting in an aspect ratio (A = d/w) of 0.309 ± 0.01 and a roughness ratio (r = 1 + 2 d/w) of 1.62 ± 0.02 [28].

### 3.2. Growth and Differentiation of N2a Cells on the Laser-Microstructured Polyethylene Terephthalate (PET) Substrates Under Static Conditions

**Figure 4:**
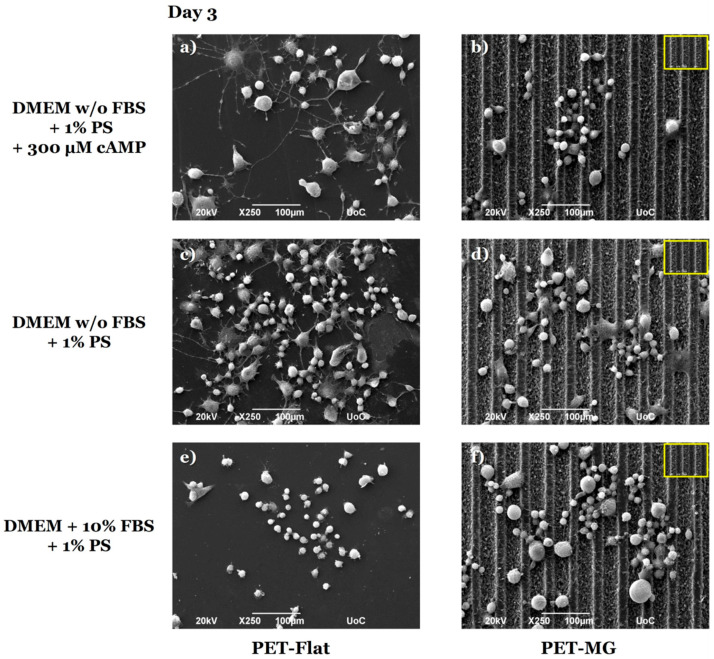
Scanning electron microscopy (SEM) images of N2a cells cultured on the PET-Flat (**a**,**c**,**e**) and PET-MG (**b**,**d**,**f**) substrates under static conditions, on the third day of culture. For the cell differentiation experiments (**a**–**d**), the culture medium (DMEM + 10% FBS + 1% antibiotic solution PS) was substituted with serum-free (w/o FBS) DMEM containing 1% antibiotic solution PS + 300 μM cAMP (**a**,**b**) or serum-free (w/o FBS) DMEM containing 1% antibiotic solution PS (**c**,**d**) after 24 h of incubation. The inset SEM images, framed by a yellow box, depict the geometry of microgrooves.

Figure 4 illustrates the morphology of N2a cells cultured on the PET-Flat (Figure 4a,c,e) and PET-MG substrates (Figure 4b,d,f) under static conditions, on the third day of culture. To perform the cell differentiation experiments (Figure 4a–d), the culture medium was substituted with either serum-free DMEM containing 1% antibiotic solution PS and 300 μM cAMP (Figure 4a,b) or serum-free DMEM containing 1% antibiotic solution PS (Figure 4c,d) after 24 h of incubation.

We observed that N2a cells exhibited good attachment and outgrowth both on the PET-Flat and PET-MG substrates (Figure 4e,f). Furthermore, we noticed that N2a cells appeared to be randomly oriented on the MGs (Figure 4f). In the differentiation experiments, we observed that N2a cells developed neurite-like extensions on the PET-Flat substrates, both with serum deprivation and with/without the addition of cAMP (Figure 4a,c). However, N2a cells failed to form neurites on the MGs and retained the rounded cell morphology of undifferentiated cells (Figure 4b,d).

**Figure 5:**
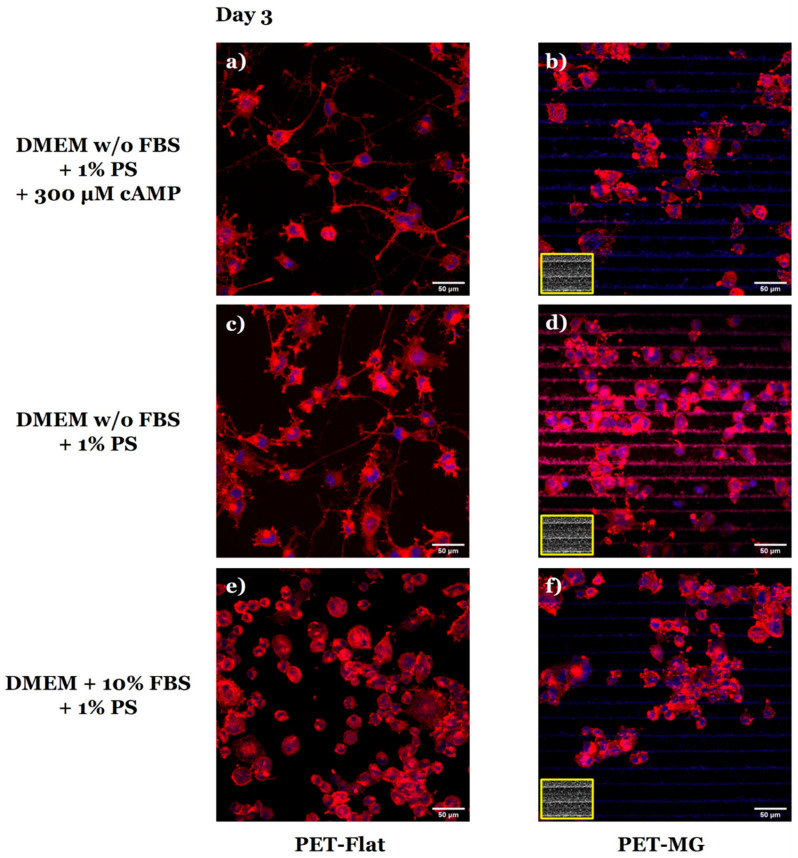
Confocal images of N2a cells cultured on the PET-Flat (**a**,**c**,**e**) and PET-MG substrates (**b**,**d**,**f**) under static conditions, on the third day of culture. The cytoskeleton of the cells is visualized with red color (Alexa Fluor^®^ 680 Phalloidin) while the nuclei with blue color (DAPI). For the cell differentiation experiments (**a**–**d**), the culture medium (DMEM + 10% FBS + 1% antibiotic solution PS) was substituted with serum-free (w/o FBS) DMEM containing 1% antibiotic solution PS + 300 μM cAMP (**a**,**b**) or serum-free (w/o FBS) DMEM containing 1% antibiotic solution PS (**c**,**d**) after 24 h of incubation. The inset scanning electron microscopy (SEM) images, framed by a yellow box, depict the geometry of microgrooves.

In agreement with the SEM images (Figure 4), the respective confocal images of N2a cells on the PET-Flat (Figure 5a,c,e) and PET-MG substrates (Figure 5b,d,f) showed that N2a cells extended neurites on the PET-Flat (Figure 5a,c), but they did not on the PET-MG substrates (Figure 5b,d).

**Figure 6:**
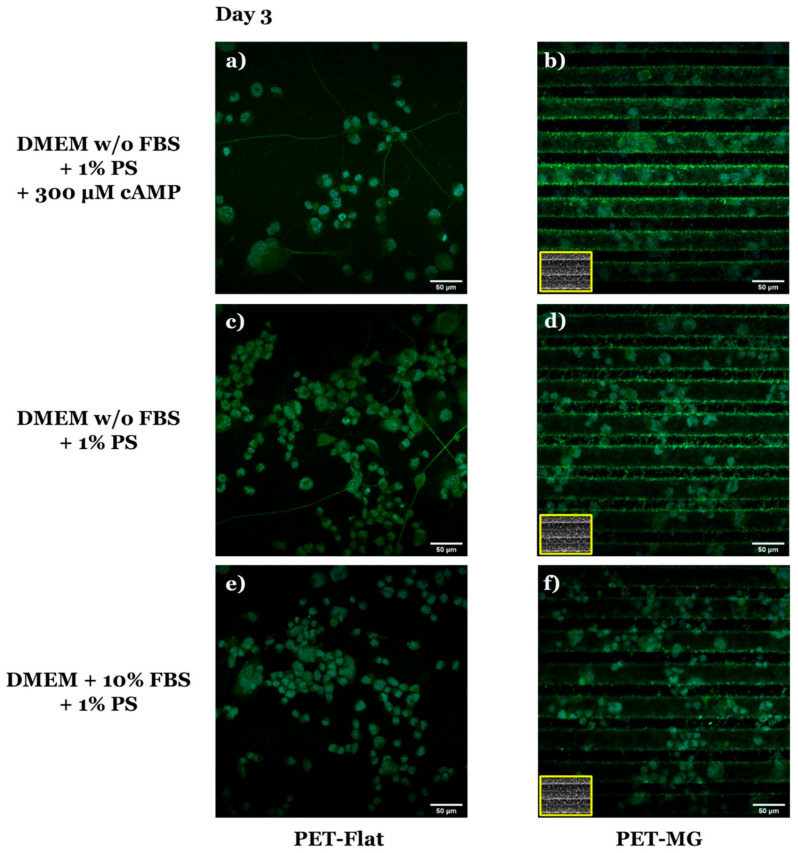
Staining of neuron-specific class III β-tubulin in N2a cells. Confocal images of N2a cells cultured on the PET-Flat (**a**,**c**,**e**) and PET-MG (**b**,**d**,**f**) substrates under static conditions, on the third day of culture. The cells were stained using the anti-mouse TUJ1 (green color) antibody. Nuclei were stained with DAPI (blue color). For the cell differentiation experiments (**a**–**d**), the culture medium (DMEM + 10% FBS + 1% antibiotic solution PS) was substituted with serum-free (w/o FBS) DMEM containing 1% antibiotic solution PS + 300 μM cAMP (**a**,**b**) or serum-free (w/o FBS) DMEM containing 1% antibiotic solution PS (**c**,**d**) after 24 h of incubation. The inset scanning electron microscopy (SEM) images, framed by a yellow box, depict the geometry of microgrooves.

The extension of neurites revealed in Figure 4a,c and Figure 5a,c, indicate the ability of N2a cells to be differentiated on the PET-Flat substrates. To verify the differentiation ability of N2a cells, the detection of class III β-tubulin, a specific neuronal marker, was studied (Figure 6). Class III β-tubulin is a microtubule protein primarily expressed in neurons, and the anti-class III β-tubulin monoclonal antibody (TUJ1) specifically detects this protein. TUJ1 is commonly used as a marker for neuronal differentiation. While class III β-tubulin is expressed in differentiated neurons, undifferentiated N2a cells can still exhibit low levels of expression, which is detectable by TUJ1 staining. This basal expression explains the TUJ1 staining observed in the undifferentiated cells on the PET-MG substrates, as evidenced by the lack of neurite outgrowth (Figure 6b,d). Additionally, these studies confirmed that the PET-Flat substrates promoted the differentiation of N2a cells into neurons, as indicated by neurite formation (Figure 6a,c). On PET-Flat substrates, neurite outgrowth was observed, and TUJ1 staining in these cells further confirmed neuronal differentiation. In contrast, the MGs inhibited differentiation, as evidenced by the rounded cell morphology (Figure 6b,d). It should also be noted that, in the presence of FBS, no differentiation was observed on the PET-Flat (Figure 6e) or PET-MG (Figure 6f) substrates, due to the absence of either serum deprivation or cAMP induction. However, TUJ1 staining was still visible in these cells, reflecting the basal expression of class III β-tubulin in undifferentiated cells.

**Figure 7:**
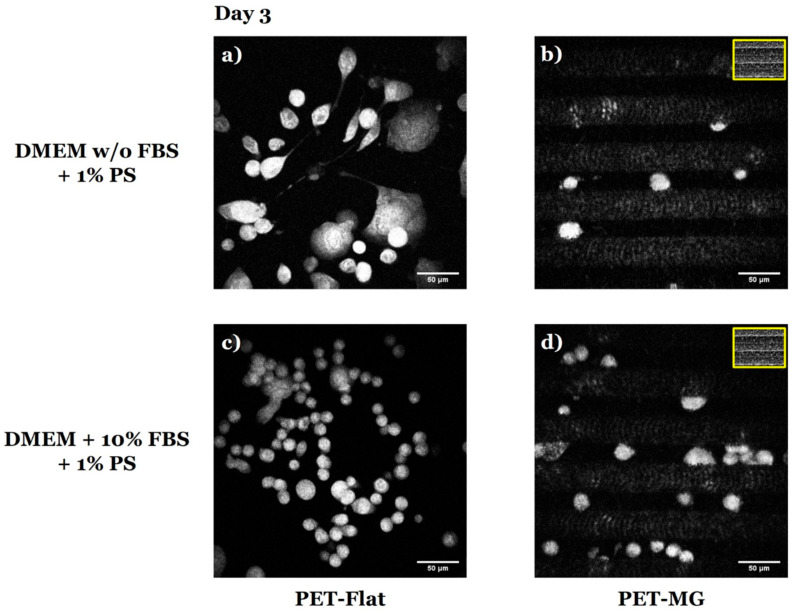
Live imaging of N2a cells cultured on the PET-Flat (**a**,**c**) and PET-MG (**b**,**d**) substrates under static conditions, on the third day of culture, using a custom-built inverted laser raster-scanning non-linear microscope. Fluorescent dye acridine orange (AO) was used. For the cell differentiation experiments (**a**,**b**), the culture medium (DMEM + 10% FBS + 1% antibiotic solution PS) was substituted with serum-free (w/o FBS) DMEM containing 1% antibiotic solution PS after 24 h of incubation. The inset scanning electron microscopy (SEM) images, framed by a yellow box, depict the geometry of microgrooves.

Furthermore, live imaging of N2a cells cultured on the PET-Flat (Figure 7a,c) and PET-MG substrates (Figure 7b,d), under static conditions, on the third day of culture, was performed using a custom-built inverted laser scanning non-linear microscope. The cell differentiation experiments (Figure 7a,b) were performed by replacing the culture medium with serum-free DMEM containing 1% antibiotic solution PS after 24 h of incubation. For the live cell imaging experiments, the fluorescent dye AO was used in order to assess cell differentiation by visualizing the cells in real-time.

Indeed, live cell imaging is an important technique for studying the phenotype of live neuron dynamics due to the fact that imaging fixed samples is unable to capture the intricacy of dynamic events that take place during the development and regeneration of the nervous system. In addition to this, live cell imaging preserves the natural environment and function of neurons, minimizes the perturbation to neural circuits, and allows more physiologically relevant data [46].

In agreement with the previous findings (Figure 4, Figure 5 and Figure 6), live cell imaging revealed that N2a cells differentiated on the PET-Flat substrate (Figure 7a), while they did not differentiate on the PET-MG substrate (Figure 7b). Specifically, on the PET-Flat substrate, differentiation was evident as the cells developed neurite-like extensions, whereas, on the PET-MG substrate, the cells maintained a rounded morphology, indicating a lack of differentiation.

### 3.3. Growth and Differentiation of N2a Cells on the Laser-Microstructured Polyethylene Terephthalate (PET) Substrates Under Dynamic Conditions

**Figure 8:**
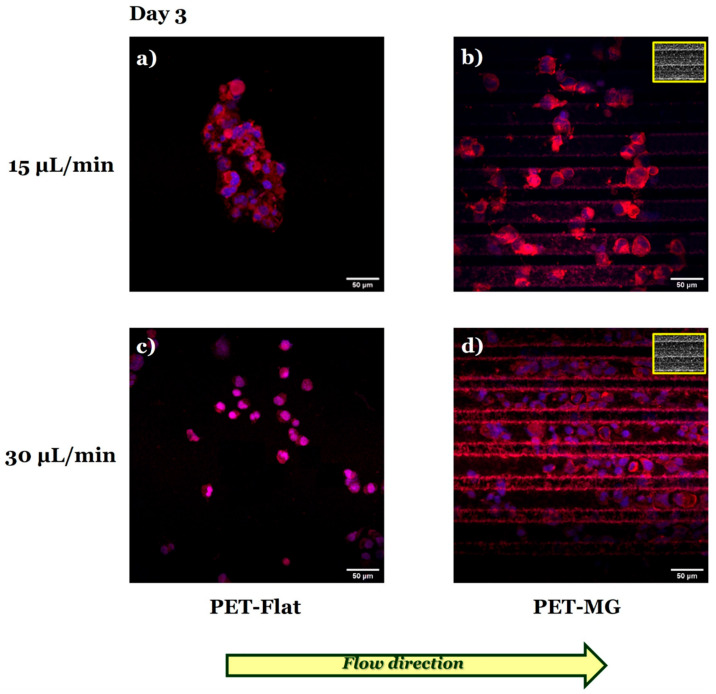
Confocal images of N2a cells cultured on the PET-Flat (**a**,**c**) and PET-MG substrates (**b**,**d**), under dynamic conditions, applying 15 (**a**,**b**) and 30 (**c**,**d**) μL/min, on the third day of culture. The cytoskeleton of the cells is visualized with red color (Alexa Fluor^®^ 680 Phalloidin) while the nuclei with blue color (DAPI). The culture medium (DMEM + 10% FBS + 1% antibiotic solution PS) was substituted with serum-free (w/o FBS) DMEM containing 1% antibiotic solution PS to perform the cell differentiation experiments after 24 h of incubation. The inset scanning electron microscopy (SEM) images, framed by a yellow box, depict the geometry of microgrooves. The yellow arrow represents the direction of the flow.

Figure 8 presents the N2a cells cultured on the PET-Flat (Figure 8a,c) and PET-MG substrates (Figure 8b,d), under dynamic conditions, applying 15 (Figure 8a,b) and 30 (Figure 8c,d) μL/min on the third day of culture. The cell differentiation experiments were performed by replacing the culture medium with serum-free DMEM containing 1% antibiotic solution PS after 24 h of incubation.

By applying both flow rates parallel to the MG length, we observed that N2a cells did not exhibit the phenotype of differentiated cells (neurite extensions) on the PET-MG substrates (Figure 8b,d), in agreement with the static cultures (Figure 4 and Figure 5). Interestingly, under dynamic conditions, N2a cells did not exhibit such a phenotype either on the PET-Flat substrates (Figure 8a,c), contradictory to the static cultures (Figure 4 and Figure 5).

**Figure 9:**
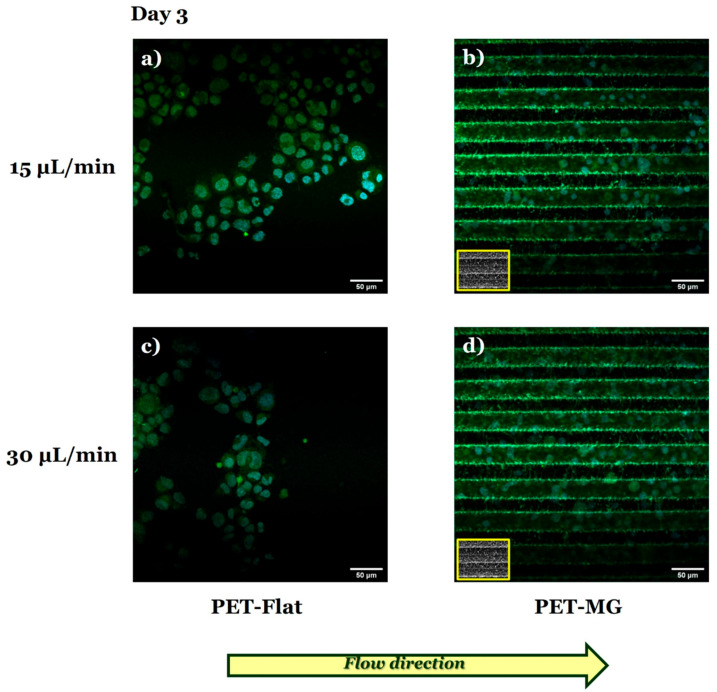
Staining of neuron-specific class III β-tubulin in N2a cells. Confocal images of N2a cells cultured on the PET-Flat (**a**,**c**) and PET-MG (**b**,**d**) substrates, under dynamic conditions, applying 15 (**a**,**b**) and 30 (**c**,**d**) μL/min, on the third day of culture. The cells were stained using the anti-mouse TUJ1 (green color) antibody. Nuclei were stained with DAPI (blue color). The culture medium (DMEM + 10% FBS + 1% antibiotic solution PS) was substituted with serum-free (w/o FBS) DMEM containing 1% antibiotic solution PS to perform the cell differentiation experiments after 24 h of incubation. The inset scanning electron microscopy (SEM) images, framed by a yellow box, depict the geometry of microgrooves. The yellow arrow represents the direction of the flow.

Further investigation of the N2a differentiation process by immunofluorescence experiments using the TUJ1 antibody (Figure 9) revealed that flow-induced shear stress (Figure 9a,c) seemed to inhibit the differentiation of N2a cells to neurons.

**Figure 10:**
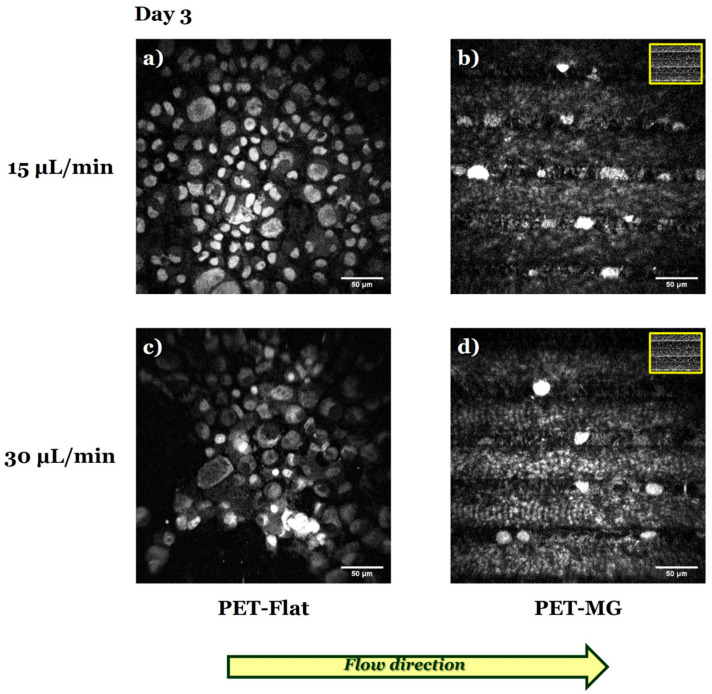
Live imaging of N2a cells cultured on the PET-Flat (**a**,**c**) and PET-MG substrates (**b**,**d**), under dynamic conditions, applying 15 (**a**,**b**) and 30 (**c**,**d**) μL/min, on the third day of culture, using a custom-built inverted laser raster-scanning non-linear microscope. Fluorescent dye acridine orange (AO) was used. The culture medium (DMEM + 10% FBS + 1% antibiotic solution PS) was substituted with serum-free (w/o FBS) DMEM containing 1% antibiotic solution PS to perform the cell differentiation experiments after 24 h of incubation. The inset scanning electron microscopy (SEM) images, framed by a yellow box, depict the geometry of microgrooves. The yellow arrow represents the direction of the flow.

Consistent with the previous findings (Figure 8 and Figure 9), live cell imaging demonstrated that N2a cells did not differentiate both on the PET-Flat (Figure 10a,c) and PET-MG substrates (Figure 10b,d) under flow-induced shear stress conditions.

## 4. Discussion

Although axons in the PNS have an inherent tendency to regenerate after injury, the regeneration often results in reduced functionality because the axons frequently become misdirected towards inappropriate targets [25,47,48]. Hence, the discovery of successful methods to study neurite outgrowth in a controllable manner, in vitro, is of great importance. Controlling neuron differentiation is crucial for neural development, disease prevention, and regenerative medicine. It maintains the balance between progenitor cells and neurons, ensuring brain function and repair while preventing disorders, tumors, and network instability. In regenerative medicine, regulation preserves progenitor cells for treating neurodegenerative diseases like Parkinson’s and Alzheimer’s. Studying factors like topography and shear stress helps optimize neural regeneration and therapeutic applications [13].

Previous studies have demonstrated that the topographical features of the culture substrate, such as grooves, fibers, ripples, and cones, play a significant role in influencing the outgrowth, orientation, and differentiation of various neuronal and glial cell types, like Schwann, PC12, and N2a cells [49,50,51,52,53,54,55,56,57,58].

However, besides topography, it has become more apparent recently that mechanical factors may also influence neurogenesis [59,60]. Shear stress has been identified as a critical factor in the host environment of regenerating axons [61]. Consequently, flow-induced shear stress could also influence neurite outgrowth. To the best of our knowledge, no studies currently exist in the literature that specifically examine the effects of shear stress, either in combination with or independent of topography on neuronal (N2a) cells, despite their widespread use as a model system to study neurite outgrowth [29,30,31,32,33,34,43].

This study aims to explore, for the first time, the combined effect of shear stress and topography on the growth and differentiation of N2a cells under dynamic culture conditions. This is achieved through continuous microfluidic flow, controlled by a precise microfluidic system that generates the applied shear stress. Building on the findings of our previous studies [27,28], which demonstrated that the MG geometry provides a favorable environment for the growth and alignment of SW10 cells, we selected this geometry to investigate the combined effect of shear stress and topography on neuronal response.

Although the effect of topography on SW10 cells has been studied thoroughly, there are limited studies on N2a cells [62,63,64,65]. Specifically, Mitra et al. [62] reported that N2a cells oriented on and along with the carbon micro-track length for micro-track width and inter-track distances of 15–30 μm, which was comparable to the cell dimension (~15 μm). Additionally, differentiated N2a cells extended neurites aligned along the micro-track axis when the track width was 15 μm. Similarly, Beduer et al. [63] showed that PDMS grooves of 20 μm width promoted the differentiation rate of N2a cells and the neurite alignment along the groove axis. On the contrary, Zhu et al. [64] demonstrated that the neurites of differentiated N2a cells were guided by silicon (Si), polyamide (PI), and transparency grooves of 300–400 nm width, 400–700 nm depth, and 1–1.2 μm period. Finally, Lee et al. [65] reported that N2a cells on silicon dioxide (SiO_2_) linear ridges with 4 μm height extended neurites following the linear micro-track. Consequently, a wide range of dimensions affecting neurite outgrowth has been proposed in the literature.

Generally, it has been shown that, on the microscale (a scale similar to that of axons), the effect of distance between topographical features on the neurons’ alignment increases as the width of the space between the cues decreases [66]. On the nanoscale, the sensitivity of neurons to nanometer-sized topographical cues is strongly evident [66]. Since cell adhesion sites, specifically focal adhesions, range from 5 to 200 nm [67], it has been proposed that cell-substrate interactions are governed by intricate mechanisms operating at this scale. In particular, it has been suggested that these interactions are more strongly influenced by nanoscale features rather than microscale ones [68,69]. Additionally, there is a notable sensitivity at the nanoscale that produces different effects on cell differentiation [66]. Indeed, previous studies have demonstrated that several neuronal markers, such as MAP2 and TUJ1, are significantly upregulated on nano-grating substrates compared to unpatterned and micro-grating substrates [70,71]. The aforementioned observations, regarding the importance of size scale, may explain in our study the omnidirectional orientation of N2a cells on the MG, as well as the inhibition of cell differentiation on them (width/spacing of MG~29 μm) (Figure 4, Figure 5, Figure 6 and Figure 7). Since the forces of values applied during nerve regeneration in vivo have not been described, based on the results of our previous work [27], we concluded to the flow rates of 15 and 30 μL/min (0.01 and 0.02 Pa, respectively) (Table 1). At higher flow rate values, N2a cells detached from the substrates.

In accordance with the static cultures, when the flow rates of 15 and 30 μL/min were applied parallel to the MG length, we observed that N2a cells remained in an undifferentiated state on the MGs (Figure 8, Figure 9 and Figure 10). Interestingly, under dynamic culture conditions (both flow rates of 15 and 30 μL/min), N2a cells did not differentiate on the PET-Flat substrates (Figure 8, Figure 9 and Figure 10) in contrast to the static cultures (Figure 4, Figure 5, Figure 6 and Figure 7). This implies that flow-induced shear stress may inhibit the differentiation of N2a cells (at least within the range of flow rates used in this study). This observation serves as an important preliminary finding, highlighting the need for further investigation of the specific mechanisms by which shear stress inhibits neuronal differentiation. Flow-induced shear stress has been reported to affect mechanoreceptors such as integrin/focal adhesions and ion channels. It also influences various cellular responses, including nitric oxide production, intracellular calcium levels, and cytoskeletal remodeling [72]. Shear stress is applied at localized points and transmitted across the cell body via cytoskeletal microstructures, triggering intracellular mechanical signaling. Consequently, it alters intracellular signaling pathways as well [25,73]. Protein screening, coupled with quantitative assessments and statistical validation, could be employed to elucidate the specific pathways involved under both static and dynamic culture conditions, providing valuable insights into the underlying mechanisms.

## 5. Conclusions

The combined effect of shear stress and topography on N2a cells’ growth and differentiation was examined. In agreement with the static cultures, it was found that, under dynamic culture conditions, N2a cells did not differentiate on the MGs. However, and more importantly, N2a cells remained undifferentiated on the PET-Flat substrates under dynamic conditions, in contrast to static cultures, suggesting that shear stress may inhibit the differentiation of N2a cells. The dynamic microenvironment system presented here offers a potential new model for studying the role of shear stress and reveals the importance of extracellular mechanical stimuli in neural cell differentiation. While both shear stress and topography influence differentiation, their combined effect provides insight into how multiple physical stimuli interact in a biomimetic environment. This approach can contribute to the design of in vitro models that better replicate physiological conditions.

## Figures and Tables

**Table 1:** Values for flow rate (Q), mean velocity [$\bar{u}$ = (4Q)/π$d^{2}$], and shear stress [σ = (6ηQ)/(b$h^{2}$)] in the microfluidic system.

Q (μL/Min)	$\bar{u}$ (m/s)	σ (Pa)
15	0.0013	0.01
30	0.0025	0.02

## Data Availability

The original contributions presented in this study are included in the article. Further inquiries can be directed to the corresponding authors.

## Abbreviations

2p-F, Two-photon fluorescence; AO, Acridine orange; BPF, Band-pass filter; BSA, Bovine serum albumin; cAMP, Cyclic AMP; CNS, Central nervous system; DAPI, 4′,6-Diamidino-2-Phenylindole; DM, Dichroic mirror; DMEM, Dulbecco’s Modified Eagle’s Medium; DRG, Dorsal root ganglion; ECM, Extracellular matrix; FBS, Fetal Bovine Serum; GDA, Glutaraldehyde; GFAP, Glial fibrillary acidic protein; GFs, Growth factors; GM, Galvanometric mirrors; hiPSCs, Human-induced pluripotent stem cells; hMSCs, Human mesenchymal stem cells; MAP2, Microtubule-associated protein 2; MGs, Microgrooves; N2a, Neuro-2a; NGF, Nerve growth factor; NSCs, Neural stem cells; O, Objective lens; PBS, Phosphate buffered saline; PC12, Pheochromocytoma; PDMS, Polydimethylsiloxane; PET, Polyethylene terephthalate; PET-MG, Polyethylene terephthalate microgrooved; PFA, Paraformaldehyde; PI, Polyamide; PLGA, Poly(lactic-co-glycolic acid); PMT, Photomultiplier tube; PNS, Peripheral nervous system; PS, Pen-Strep; PTFE, Poly(tetrafluoroethylene); RGCs, Radial glial cells; SCB, Sodium cacodylate buffer; SEM, Scanning electron microscopy; Si, Silicon; SP, Sample plane; SPF, Short-pass filter; SW10, Schwann; UV, Ultraviolet.
